# Thigh-Worn Accelerometer Data in the Maastricht Study

**DOI:** 10.1093/geroni/igaa057.2756

**Published:** 2020-12-16

**Authors:** Annemarie Koster

**Affiliations:** Maastricht University, Maastricht, Limburg, Netherlands

## Abstract

This study describes the use of thigh-worn accelerometers to collect high-quality data on sedentary time and physical activity in the Maastricht Study, a prospective cohort study in the Netherlands. Data have been collected in 9000 participants, aged 40-75 years, 49% women, and 25% has type 2 diabetes by design. All participants were asked to wear an activPAL accelerometer for 7 days. Percentage sedentary, standing and stepping time of waking time were calculated. Participants had on average 6.4 valid days (≥14h of monitoring) and 90% wore the device >4 days. Men spent significantly more time sedentary than women (63.3±10.3% versus 57.0±10.1%); standing and stepping time were significantly higher in women (30.1±7.9%; 12.9±4.4%) than in men (24.7±7.4%; 12.1±4.6%). Sedentary time significantly increased with increasing age while standing time decreased; no clear age-gradient in stepping time was found.

